# Sintilimab plus fruquintinib with or without radiotherapy for third-line treatment of colorectal cancer with liver metastases: study protocol for a randomized controlled, multicenter phase II trial

**DOI:** 10.3389/fimmu.2026.1622828

**Published:** 2026-05-12

**Authors:** Zhenyu Ma, Kunli Zhu, Fang Shi, Rui Feng, Shumei Jiang, Xue Dou, Jing Liu, Zuoxing Niu, Jinbo Yue

**Affiliations:** 1 Shandong University Cancer Center, Cheeloo College of Medicine, Shandong University, Jinan, Shandong, China; 2 Shandong Cancer Hospital and Institute, Shandong First Medical University and Shandong Academy of Medical Sciences, Jinan, Shandong, China

**Keywords:** fruquintinib, immunotherapy, liver metastases, liver-directed radiotherapy, metastatic colorectal cancer, sintilimab

## Abstract

**Background:**

Metastatic colorectal cancer (mCRC) has limited treatment options beyond the second-line. Accumulating evidence has reported that liver metastases harbor a greater abundance of immunosuppressive cells relative to primary tumors. However, about 70% of mCRC patients have liver metastases, which worsen prognosis and reduce immunotherapy benefits. Local therapies, such as liver-directed radiotherapy, have shown promise in reducing tumor burden and improving survival.

**Methods:**

This phase II trial aims to evaluate the efficacy and safety of sintilimab combined with fruquintinib, with or without liver-directed radiotherapy, versus fruquintinib alone in the third-line treatment of colorectal cancer with liver metastases (CRCLM). Patients aged 18–75 years with microsatellite stable CRCLM who have received first- and second-line therapies will be randomized 3:3:2 into intervention, control, and standardized treatment groups. Patients in the intervention group will be given sintilimab plus fruquintinib in combination with liver-directed radiotherapy. For liver oligometastases, stereotactic body radiation therapy (SBRT) will be performed with a dose of 40–50 Gy/5 F, 60–70 Gy/10 F, or 65 Gy/13 F. For multiple liver metastases, SBRT plus low-dose radiation therapy (LDRT) will be performed. The appropriate lesions will be selected to receive SBRT with a dose of 40–50 Gy/5 F, 60–70 Gy/10 F, or 65 Gy/13 F, and the remaining liver lesions will undergo LDRT at a total dose of 1–10 Gy with 0.5-2.0 Gy/F. Patients in the control group will be treated with sintilimab plus fruquintinib, and patients in the standardized treatment group will be treated with fruquintinib only. The primary endpoint is PFS and secondary endpoints are objective response rate, disease control rate, OS, and safety.

**Discussion:**

We hypothesize that combining sintilimab with liver-directed radiation therapy on the basis of fruquintinib will enhance disease control and prolong survival compared to treatment with fruquintinib plus sintilimab alone.

**Clinical Trial Registration:**

clinicalTrials.gov, identifier (NCT06356584).

## 1 Introduction

Early symptoms of colorectal cancer (CRC) are not obvious, and 25% of patients have distant metastasis at diagnosis, which cannot be cured by surgery alone (1). For metastatic CRC (mCRC), the progression-free survival (PFS) is about 10–12 months for first-line and 6 months for second-line therapy (2, 3). After failure of first- and second-line treatments, third-line and beyond therapies remain suboptimal. Currently, third-line therapies of mCRC recommended by major guidelines include fruquintinib, regorafenib, and trifluridine-tipiracil (TAS-102) with or without bevacizumab (4–6). Although extensive data for third-line treatment of mCRC have been published, the results show that the prognosis remains poor, with a median PFS (mPFS) of only 3.2-5.6 months (7–12). There is an urgent need to find novel therapies.

At present, immune checkpoint inhibitors (ICIs), such as programmed death-1 (PD-1) monoclonal antibodies, have achieved great success in clinical practice; however, for mCRC, the population benefiting from ICIs is still limited to about 5% of patients with high microsatellite instability (MSI-H) (13). The microsatellite stable (MSS) type, which accounts for 95% of mCRC, shows limited response to immunotherapy. Combination regimens are a major focus of immunotherapy research, and immunotherapy combined with targeted therapy holds great promise (14). Clinical studies have shown that targeted drugs combined with ICIs hold promising prospects for improving survival outcomes for MSS mCRC patients (15–17).

Liver metastases significantly impair the response of mCRC to immunotherapy. Yu et al. showed that liver metastases can exploit immune tolerance mechanisms, leading to CD8^+^ T cell depletion (18). Lee et al. described another mechanism—the regulation of regulatory T cell recruitment and the activation and modulation of CD11b^+^ monocytes at the primary tumor site (19). Multiplex immunohistochemistry analysis within the REGONIVO and LENPEM cohorts revealed that liver metastases have more immunosuppressive cells and fewer CD8^+^ T cells compared to primary tumors (20). Adding local therapies to systemic therapy can effectively reduce tumor burden and may contribute to better survival (21, 22). Radiotherapy, with high local control rates and a favorable safety profile, is one of the most important local treatment modalities and provides substantial clinical benefits to patients. Notably, liver-directed radiotherapy combined with immunotherapy can restore immune cell function, enhance anti-cancer immunity, and increase sensitivity to anti-PD-L1 therapy (18).

Herein, the aim of this study is to evaluate the efficacy and safety of fruquintinib plus sintilimab combined with liver-directed radiotherapy versus fruquintinib plus sintilimab for third-line treatment of CRC with liver metastasis (CRCLM).

## 2 Methods

### 2.1 Objectives

The aim of this study is to evaluate the efficacy and safety of sintilimab in combination with fruquintinib with or without liver-directed radiotherapy for the third-line treatment of CRCLM.

### 2.2 Study design

This study is a randomized, controlled, multicenter phase II clinical trial, which will be conducted at the Shandong Cancer Hospital and Institute. The study has been authorized by the ethics committee of Shandong Cancer Hospital and Institute (No. SDZLEC2024-078-01) and all patients will provide written informed consent before enrollment. The trial has been registered on the ClinicalTrials.gov under the registration number NCT06356584 (June 21, 2025).

### 2.3 Study population

#### 2.3.1 Inclusion criteria

1. Men or women between the ages of 18 and 75 years old.
2. Eastern Cooperative Oncology Group Performance Status (ECOG PS) Score from 0 to 2.
3. Have histopathologically confirmed colorectal adenocarcinoma with liver metastases according to the *American Joint Committee on Cancer 8th edition* classification of malignancies.
4. *RAS* and *BRAF* gene mutant or wild types, MSS type.
5. Previously received standard first- and second-line systemic anti-cancer therapy.
6. Have at least one measurable lesion as defined by RECIST 1.1 standards.
7. Tumor samples can be obtained for biomarker evaluation.
8. Expected survival ≥ 3 months.
9. Normal major organ function two weeks prior to enrollment.

#### 2.3.2 Exclusion criteria

1. Diagnosis of a malignancy other than CRC within the past 3 years (excluding radically treated basal cell carcinoma of the skin, squamous epithelial carcinoma of the skin, and/or radically resected carcinoma *in situ*).
2. MSI-H tumors.
3. History of treatment with fruquintinib or regorafenib and immunotherapy.
4. Liver-directed radiotherapy within 2 weeks prior to enrollment.
5. Treated with other investigational agents or with investigational devices within 4 weeks prior to enrollment.
6. Being allergic to active ingredients or excipients of sintilimab or fruquintinib.
7. Central nervous system metastases and/or carcinomatous meningitis.
8. Pregnant or breastfeeding women.
9. Any serious or uncontrolled systemic disease.

### 2.4 Randomization and blinding

All included patients will be randomized into intervention, control, and standardized treatment groups in a 3:3:2 ratio using a computer-generated random sequence by a clinical research assistant. This study is an open-label trial, so participants, care providers, and investigators will not be blinded.

### 2.5 Interventions

**Figure 1:**
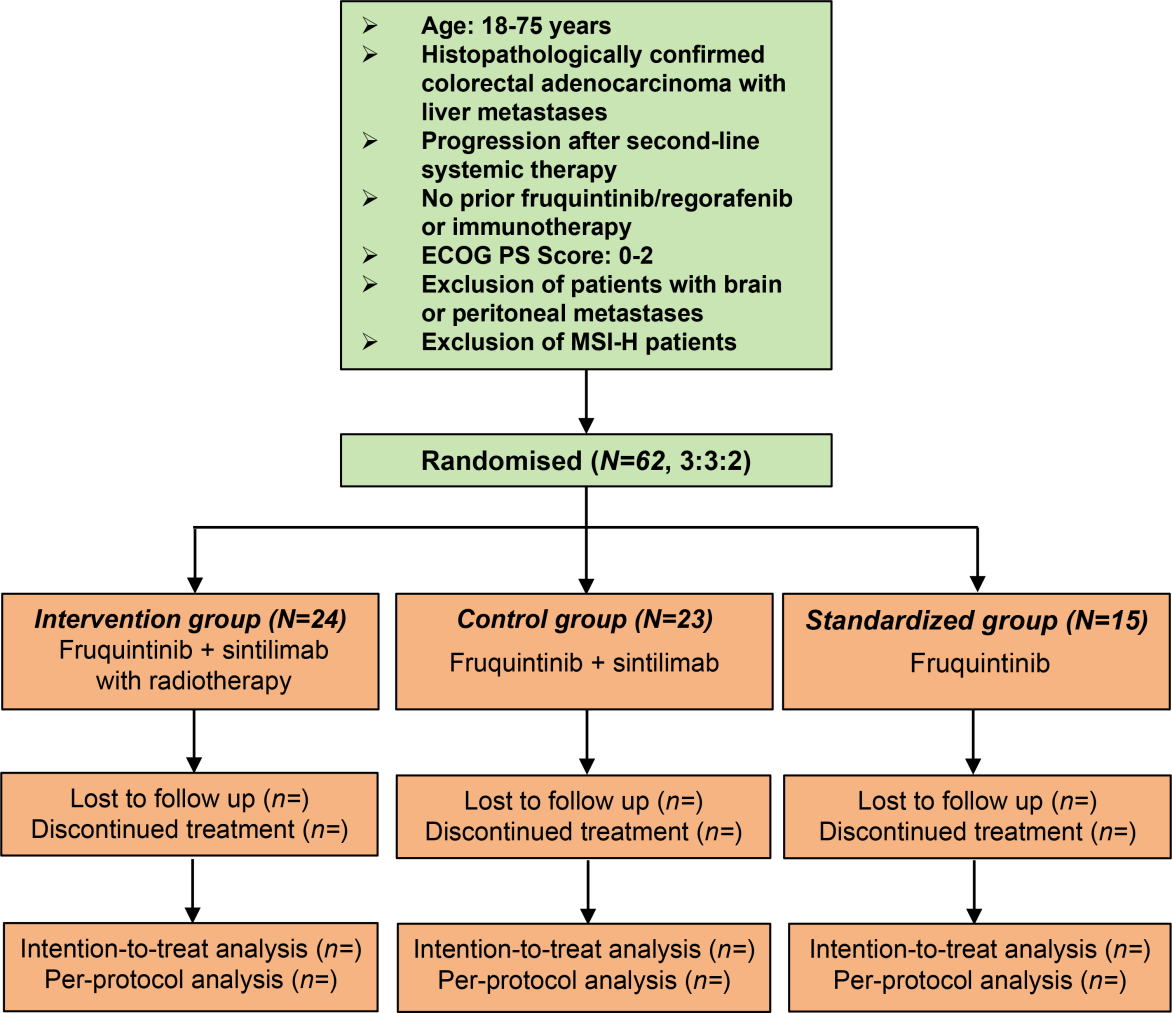
Study flow of the present study. In this phase II study, we will assess the efficacy and safety of sintilimab and fruquintinib combined with liver-directed radiation therapy versus sintilimab and fruquintinib for the third-line treatment of colorectal cancer with liver metastases.

**Figure 2:**
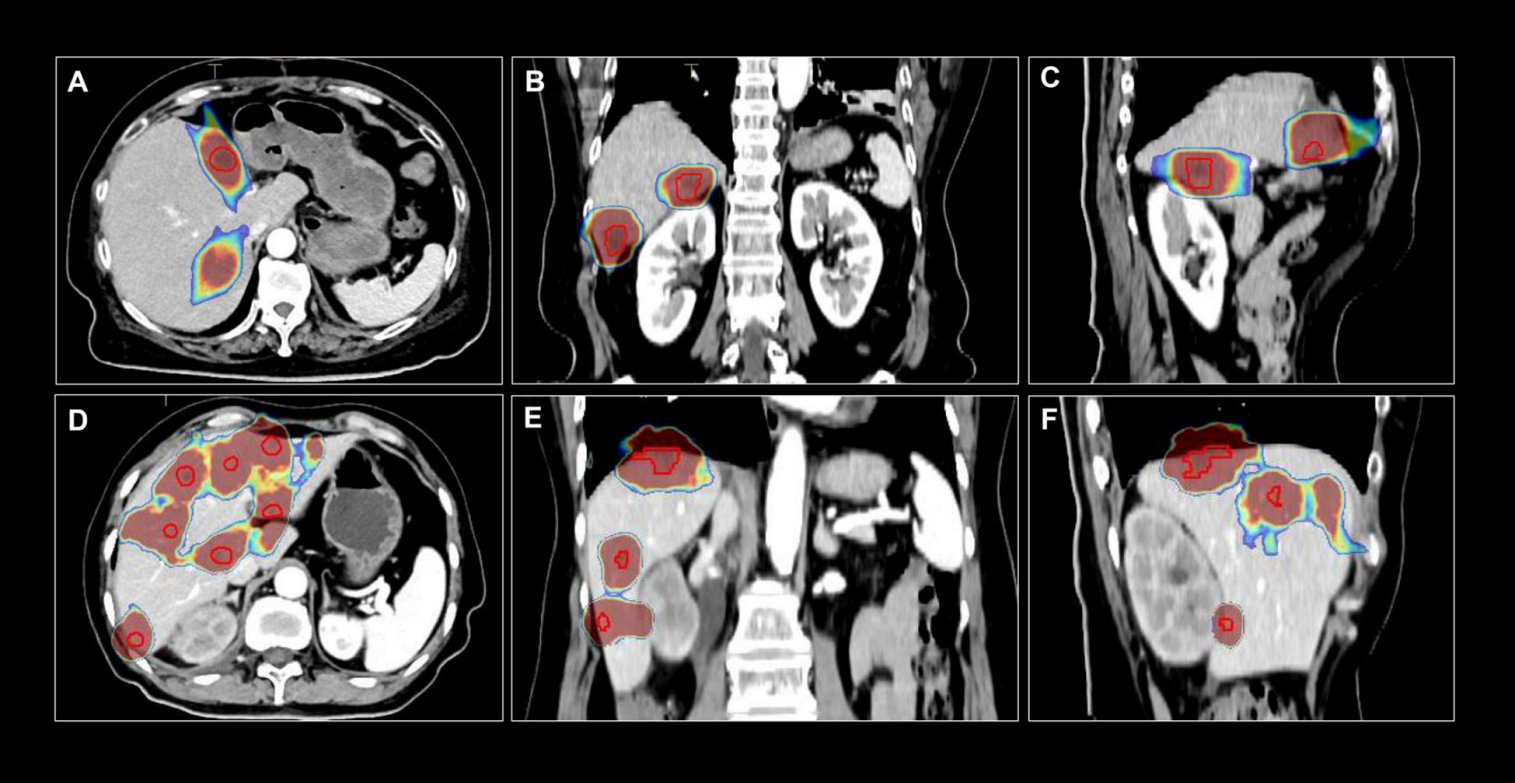
An example of target area delineation and radiation dose distribution for two patients. The top row **(A–C)** represents a patient with liver oligometastasis receiving 40 Gy/10 F irradiation, with the images displayed in axial, coronal, and sagittal planes. The bottom row **(D–F)** represents a patient with multiple liver metastases receiving 10 Gy/5 F irradiation, with the images displayed in axial, coronal, and sagittal planes.

The study flow is presented in **Figure 1**. Patients in the intervention group will be given sintilimab (200 mg, IV, D1, once every 3 weeks) plus fruquintinib (5 mg, po, D1-14, once every 3 weeks) in combination with liver-directed radiotherapy. A 21-day treatment cycle of sintilimab (200 mg, IV, D1, once every 3 weeks) will be given intravenously on day 1 of each cycle, and fruquintinib will be given on days 1 through 14 of each cycle. The radiologist will determine the radiation division pattern based on the location and volume of the liver metastases. For liver oligometastases, stereotactic body radiation therapy (SBRT) will be performed with a dose of 40–50 Gy/5 F, 60–70 Gy/10 F, or 65 Gy/13 F. For multiple liver metastases, SBRT plus low-dose radiation therapy (LDRT) will be performed. The appropriate lesions will be selected to receive SBRT with a dose of 40–50 Gy/5 F, 60–70 Gy/10 F, or 65 Gy/13 F, and the remaining liver lesions will undergo LDRT at a total dose of 1–10 Gy with 0.5-2.0 Gy/F. One or more appropriate lesions will be selected by the physician based on the surrounding organs at risk. Extrahepatic lesions will not receive radiation therapy. Liver radiotherapy will be administered within 1 week of initiating immunotherapy. For patients receiving a 5-fraction regimen, the volume receiving ≥15 Gy will be limited to <700 cc and the Dmean will be <16 Gy. For those treated with 10 or 13 fractions, the volume receiving ≥24 Gy will be limited to <700 cc and Dmean will be <24 Gy for patients with Child-Pugh class A, and the limits will be V18Gy <700 cc and Dmean <18 Gy for patients with Child-Pugh class B. **Figure 2** shows an example of target area delineation and radiation dose distribution for two patients.

Patients in the control group will be treated with sintilimab plus fruquintinib. A 21-day treatment cycle of sintilimab (200 mg) will be given intravenously on day 1 of each cycle, and a 21-day treatment cycle of fruquintinib (5 mg) will be given orally on days 1 through 14 of each cycle. Patients in the standardized treatment group will be treated with fruquintinib (5 mg) only, and a 21-day treatment cycle of fruquintinib will be given on days 1 through 14 of each cycle.

For all included patients, CT scans will be repeated every 12 weeks during the treatment to evaluate the efficacy, and the regimen will be continued if the response is evaluated as complete response (CR), partial response (PR), stable disease (SD), or non-progressive disease (PD). Sintilimab is administered for no more than 2 years. No dose adjustments of sintilimab are permitted throughout the study. Fruquintinib is continued until toxicity is intolerable, PD, or death.

### 2.6 Follow-up

A safety follow-up will be conducted within 30 days (± 7 days) after the last dose of the study drug, or prior to initiating a new anti-cancer therapy, whichever occurs first. After safety follow-up, subjects will enter survival follow-up, where subjects will be contacted every 90 days (± 7 days) to obtain comprehensive information regarding survival status and any subsequent systemic anti-cancer therapies. Long-term follow-up will continue until the end of the study, death of the subjects, loss to follow-up, withdrawal of consent, or early termination of the study at the option of the sponsor. The details of the patient assessment schedule are described in **Supplementary Table 1**.

### 2.7 Tumor response assessment

#### 2.7.1 Baseline tumor imaging

The first tumor imaging at screening must be conducted within 28 days prior to the first administration of the study drug and will serve as the basis for the oncology assessment during the screening period. The imaging typically includes contrast-enhanced CT or MRI of the chest and abdomen. Pelvic CT or MRI, cranial MRI, and whole-body bone imaging are also required at baseline. PET/CT is also acceptable as a screening method for baseline evaluation.

#### 2.7.2 Tumor imaging during the study

Tumor imaging will be evaluated every 12 weeks (± 7 days) starting from the first dose. Unscheduled imaging evaluations will be conducted at any time if the subject experiences clinical disease instability during the study.

#### 2.7.3 Tumor imaging at the end of treatment and during the follow-up

For subjects who complete treatment or discontinue treatment for reasons other than disease progression, the tumor imaging assessment should be performed. Subsequent imaging assessments should continue at the protocol-specified time points until one of the following occurs: initiation of a new anti-cancer therapy, objective disease progression, death, or study completion, whichever comes first.

### 2.8 Toxicity monitoring and dose modification

Each subject will be assessed for adverse events (AEs) during the study or follow-up, which will be graded and documented according to National Cancer Institute General Terminology for Adverse Events (NCI-CTCAE), version 5.0. All AEs of unknown etiology occurring during the treatment should be evaluated for treatment-related AEs. An AE is defined as any adverse medical event that occurs in a clinical research subject from the time of signing the informed consent form, whether or not causally related to the study drug. A serious adverse event (SAE) is defined as an adverse medical event that meets at least one of the following criteria:1) Deaths, excluding deaths due to disease progression for study indications; 2) Life-threatening, meaning that the subject is at risk of death when this AE occurs; 3) Hospitalization or extended hospitalization; 4) Permanent or severe disability/incapacity; 5) Congenital anomalies/birth defects; 6) Other significant medical events, defined as events that jeopardize the subject or require medical intervention to prevent any of the above.

Dose-limiting toxicities are defined as Grade ≥4 hematologic toxicities, Grade ≥3 non-hematologic toxicities, and any toxicity requiring discontinuation of either fruquintinib or sintilimab for ≥21 days. Before each treatment cycle, toxicity will be assessed through patient history, physical examination, and laboratory evaluations (including complete blood count, blood biochemistry, coagulation, thyroid function, urinalysis, serum tumor markers). Notably, for subjects whose ALT or AST increases by >3× ULN, or increases by >2× baseline level, blood biochemistry tests should be performed per institutional standard practice (8). For subjects with normal baseline ALT, AST, or total bilirubin (TBIL), grade 2 elevations of AST, ALT (3–5 × ULN), or TBIL (1.5–3 × ULN); and for subjects with baseline ALT, AST, or TBIL > ULN, elevations of AST, ALT, or TBIL of ≥50% from baseline (meeting grade 2 criteria) with a duration of <7 days: dosing should be interrupted until the event improves to grade 0–1 or baseline level, after which dosing may be resumed. For subjects with normal baseline ALT, AST, or TBIL, grade 3 or 4 elevations of AST, ALT (>5 × ULN), or TBIL (>3 × ULN); and for subjects with baseline ALT, AST, or TBIL > ULN, elevations of AST, ALT, or TBIL of ≥50% from baseline (meeting grade 3 or 4 criteria) with a duration of ≥7 days: permanent discontinuation of the study drug is required.

For AEs related to fruquintinib, the dose of fruquintinib will be reduced to 3 mg/day for Grade ≥3 toxicity, and further reduced to 2 mg/day if necessary (8, 23). After AEs resolve to Grade 0–1 or to baseline levels, drug administration as scheduled will be resumed. Close monitoring for proteinuria and hypertension will be performed per protocol throughout the study. Importantly, for proteinuria of grade 2 or higher (≥2g/24h), fruquintinib will be temporarily withheld until the level decreases to <1g/24h, after which treatment can be resumed at a reduced dose. Permanent discontinuation will be required in cases of nephrotic syndrome or if proteinuria does not recover to <1g/24h. Hypertension will be managed according to American Heart Association guideline. Grade 3 hypertension will necessitate treatment interruption; upon recovery to grade 1, therapy can be restarted at a lower dose. Any grade 4 hypertension will result in permanent treatment discontinuation. Dose adjustments of sintilimab are not permitted in this trial. To manage intolerable AEs, sintilimab administration will be withheld until toxicity recovers to Grade 0–1 or to baseline levels. The maximum permitted interruption of sintilimab due to corticosteroid use for managing immune-related adverse events will not exceed 12 weeks.

Investigators must ensure that patients receive the prescribed radiation dose as defined in the protocol. If a treatment-related adverse event (TRAE) necessitates an interruption of radiotherapy, the pause should not exceed two weeks. If an interruption extends beyond two weeks due to TRAE, the decision to resume radiotherapy must be made following careful evaluation by investigators. In such cases, the patient’s treatment plan will be considered a major protocol deviation, and the patient will be regarded as a dropout. If radiotherapy is interrupted due to TRAE, administration of the study drug should be suspended concurrently. To facilitate treatment completion, appropriate prophylactic measures and supportive nutritional care are recommended. Treatment may resume once the AE recovers to CTCAE Grade 2 or lower.

### 2.9 Outcome definitions

#### 2.9.1 Primary outcome

The primary outcome of this study is PFS, defined as the time from treatment initiation to first radiological disease progression according to the Response Evaluation Criteria in Solid Tumors version 1.1 (RECIST v1.1) or death, whichever occurs first. The radiologic evaluation will be performed every 12 weeks (± 7 days) by unblinded investigators and centrally by an independent group of radiology experts, beginning after treatment initiation.

#### 2.9.2 Secondary outcomes

1. Objective response rate (ORR), defined as the proportion of subjects with a CR or PR by RECIST v1.1;
2. Disease control rate (DCR), defined as the proportion of subjects with a CR, PR, or SD by RECIST v1.1;
3. Overall survival (OS), defined as the time from treatment initiation to death from any cause;
4. The incidence, severity, and association of any AEs with study drugs, treatment-emergent adverse events (TEAEs), SAEs, and immune-related adverse events (irAEs).

### 2.10 Exploratory analyses

To explore biomarkers that can predict efficacy, we will collect biological samples, including 10–20 ml of blood, 10–20 ml of stool samples, and 10–20 tumor tissue sections, to support the analysis of cellular components. Biomarkers tested will include but are not limited to: tumor microenvironment, tumor mutation burden, stimulator of interferon genes, immuno-negative genes, immune-positive genes, hyperprogression-related genes, genes related to DNA damage repair pathways, and subpopulations of peripheral blood mononuclear cells.

### 2.11 Data collection and monitoring

The raw data of the study will be recorded and collected based on medical records or specific worksheets. All data will be cross-checked independently by two investigators. The researchers will retain all research records and original documents for 5 years after the study concludes. Security and environmental risk issues should be considered when preserving documents. The researchers will allow the relevant regulatory agencies direct access to all study-related documents, including participants’ medical records.

### 2.12 Discontinuation/withdrawal/loss to follow-up

In the event of participant withdrawal or study termination, investigators will continue to collect data on AEs and survival status. For subjects who withdraw but remain eligible for follow-up, all procedures outlined in **Supplementary Table 1** will be performed. Participants who discontinue the study for reasons other than disease progression must undergo an imaging assessment upon treatment completion. If a scheduled follow-up visit is missed, the investigator will make attempts to contact the participant to reschedule the appointment and will document the outcome of these efforts.

### 2.13 Statistical analysis

#### 2.13.1 Sample size estimation

As reported in the literature (15), the mPFS of CRCLM subgroup in the control group is 1.8 months (regorafenib + nivolumab). The mPFS in the intervention group is estimated to be 3.7 months with a hazard ratio (HR) of 0.5. With a one-sided significance level of 0.05 (power of test of 80%), case enrollment of 18 months and follow-up of 12 months, and assuming a 5% dropout rate, a 1:1 enrollment ratio for the intervention and control groups will require sample sizes of 24 and 23 cases, respectively. In addition, a standardized treatment group with a sample size of 15 cases will be set up, totaling 62 cases. These three groups will be randomly enrolled in a 3:3:2 ratio. It is worth noting that the standardized treatment group is mainly used for descriptive comparisons and safety references, and is not included in the scope of the main hypothesis tests. The comparison between the intervention group and the control group is designated as the primary analysis.

#### 2.13.2 Statistical analysis methods

Clinical characteristics and safety outcomes will be summarized using descriptive statistics. Unless otherwise stated, continuous data will be statistically described using mean (± standard deviation) or median (minimum, maximum). Categorical data will be statistically described using frequencies (percentages). Cox proportional hazard models will be used for univariate and multivariate survival analyses, with HRs and 95% confidence intervals (CIs) calculated. Subgroup analyses will be conducted by the characteristics of patients and liver metastases (liver oligometastases or multiple liver metastases). Both the intention-to-treat (ITT) population and the per-protocol population will be used for efficacy and safety analyses. The worst-case imputation and complete-case analysis will be performed as sensitivity analyses to handle the missing data and dropouts (24). The SAS 9.2 (or higher) will be used for statistical analyses, using a one-sided 0.05 hypothesis test of superiority, and 95% CIs and *P* values will be reported for group comparisons.

## 3 Discussion

For mCRC patients, the primary goal of third-line therapy is to prolong disease control and maintain quality of life and performance status, and more than 50% of mCRC patients receive third-line therapy. However, approximately 90% of previously treated mCRC patients lack therapeutically available targets. Consequently, for mCRC patients, effective third-line and beyond therapies are relatively scarce.

Fruquintinib is a highly selective oral TKI. The FRESCO study demonstrated that for patients with mCRC who failed second-line therapy, the fruquintinib group had a significantly prolonged mOS compared with the placebo group (25). However, for third-line and beyond treatment of mCRC patients, targeted therapy alone does not provide satisfactory survival benefit to patients. Targeted therapy in combination with immunotherapy has been much explored in clinical studies and holds promising prospects. Importantly, about 70% of mCRC patients have liver metastases, and mCRC patients with liver metastases derive significantly less clinical benefit from immunotherapy compared to patients without (15, 26–28). In mice, liver metastasis can recruit immunosuppressive macrophages that promote antigen-specific T cell apoptosis in the liver. This can lead to systemic loss of T cells and can reduce the efficacy of immunotherapy (18).

In the era of cancer immunotherapy, the synergistic effect of radiotherapy and immunotherapy has been confirmed by several studies. Preclinical studies have shown that radiotherapy can clinically control hepatic tumors and stimulate anti-tumor immunity (29–31). In the liver metastasis model of CRC, liver-directed radiotherapy has the potential to remodel the hepatic immune microenvironment, effectively prevent the hepatic siphoning of T cells, and thereby restore the immunotherapeutic effect (32). Liver-directed radiotherapy significantly enhanced the proliferation and IFN-γ production of subcutaneous tumor dLN CD8^+^ and CD4^+^ T cells (18). This result was further enhanced in mice receiving combination therapy. Anti-PD-L1 did not increase the number of T cells in subcutaneous tumors in mice with liver metastases, and liver-directed radiotherapy alone did not modulate the number of T cells. However, the combination of the two significantly increased T cell infiltration into subcutaneous tumors (18). Therefore, we will utilize a time window of one week after the initiation of immunotherapy to implement liver-directed radiotherapy to inhibit the “phagocytosis” of activated T cells by liver metastatic lesions and the induction of apoptosis in these cells.

LDRT is often overlooked in clinical practice. This low-dose modality, despite having a relatively weak direct cytotoxic effect on tumor cells, can stimulate immunity by altering the tumor microenvironment and enhance the effect of immunotherapy. Specifically, LDRT can trigger the activation of tumor immunotherapy. It has the capacity to reprogram the tumor microenvironment from an immunosuppressed state to an immunostimulated one (33). Moreover, LDRT may eliminate the immune privilege characteristic of the liver, allowing immunotherapy to function more effectively. In Herrera et al.’s study, the proportion of CD8^+^ T cells at different radiation doses was observed, and 1 Gy was identified as the optimal low-irradiation dose (34). They also discovered that LDRT reversed tumor immune desertification and resistance to immunotherapy. It has been shown that the combination of high-dose and low-dose radiotherapy can modulate the immune microenvironment and significantly activate immunity, as well as enhance leukocyte infiltration in the tumor, without changing the total radiation dose (35).

However, these promising disease control and survival outcomes reported here are based on studies with small sample sizes, raising concerns about selection bias. It is necessary to conduct larger-scale prospective trials to further validate these findings. For safety considerations, patients with central nervous system metastases were excluded from this trial. However, given that about 3-5% of mCRC patients present with brain metastases, this exclusion criterion inevitably will limit the generalisability of the findings to the broader mCRC population. Similarly, patients without MSI-H will also limit the generalisability. Future studies may consider exploring the safety and efficacy of this regimen in this specific subgroup. Furthermore, the application of a one-sided superiority test will increase the risk of type I error.

In conclusion, this study is a randomized, controlled, multicenter phase II clinical study exploring the efficacy and safety of combining sintilimab with or without liver-directed radiation on the basis of fruquintinib in the third-line treatment for CRCLM. We expect that the combination of sintilimab and radiotherapy will improve disease control and prolong the survival time of patients compared to fruquintinib plus sintilimab alone. This study will be published in a peer-reviewed journal, providing strong evidence-based medical proof for third-line treatment of CRCLM.
