# Supplementary material for: Sintilimab plus fruquintinib with or without radiotherapy for third-line treatment of colorectal cancer with liver metastases: study protocol for a randomized controlled, multicenter phase II trial
Source: Front Immunol. 2026 May 12;17:1622828. doi: 10.3389/fimmu.2026.1622828 (PMC13201437; doi:10.3389/fimmu.2026.1622828)
Supplement: Supplementary file 1 [file DataSheet1.pdf]

Supplementary Material

Table S1. Patient assessment schedule.

**Item**	**Screening Period**	**Treatment period (1 treatment cycle every 21 or 28 days)**			**Safety follow-up[4]**	**Survival follow-up[5]**
		**C1/D1**	**C2/D1**	**Cn/D1**		
Days	-28 days~-1 day	1 day	22 days or 29 days	21 (n-1) days +1 day or 28 (n-1) days +1 day	30 days after last administration	Every 90 days
Time window (days)	NA	+3 days	±3 days	±3 days	±7 days	±7 days
**General research process**						
Written informed consent	X					
Inclusion/exclusion criteria	X					
Demographic data/tumor/other medical history	X					
Previous/concomitant medications	X	X	X	X	X	
Vital signs	X	X	X	X	X	
Weight/height	X	X	X	X	X	
Full physical examination	X	X	X	X	X	
ECOG score	X	X	X	X	X	
12-lead electrocardiogram	X		X	X	X	
**Laboratory test**						
Blood routine test	X		X	X	X	
Blood biochemistry test	X		X	X	X	
Urine routine test	X		X	X	X	

Stool routine test	X		X	X	X	
Coagulation function test	X		X	X	X	
Pregnancy test	X					
Thyroid function	X		X	X	X	
Myocardial enzyme spectrum	X		X	X	X	
Tests for hepatitis B, hepatitis C and HIV	X					
**Security monitoring and survival**						
Adverse events	X	X	X	X	X	X
Subsequent anti-cancer therapy						X
Survival status						X
**Efficacy assessment**						
Tumor imaging assessment	X	X (every 12 weeks)			X	
**Treatment**[1]						
Sintilimab		X	X	X		
Targeted drugs (fruquintinib/regorafenib)		X	X	X		
Radiotherapy		X[6]				
**Biomarker exploration**						
Fresh tumor tissue[2]	X	X (collected at disease progression)				
Whole blood[3]	X	X (every 12 weeks)				
Faecal samples[3]	X	X (every 12 weeks)				

[1] Sintilimab (200 mg, Q3W, Day 1) will be administered continuously until disease progression or death, with a maximum treatment duration of 2 years. Experimental group: Fruquintinib (5 mg, orally, D1-14, every 3 weeks); Regorafenib (160 mg, orally, D1-14, every 3 weeks). Control group: Fruquintinib (5 mg, orally, D1-21, every 4 weeks); Regorafenib (160 mg, orally, D1-21, every 4 weeks). Targeted therapy will continue until disease progression or death.

[2] Tumor tissue samples will be collected during the screening period (allowing collection of samples within 6 months prior to treatment initiation) and at the time of disease progression. The

tumor tissue may be formalin-fixed paraffin-embedded (FFPE) tissue blocks or unstained tumor specimen slides (freshly obtained samples are preferred). Tissue samples should contain at least 20% tumor cells. FFPE samples: 15-20 slides. Fresh tissue samples: ≥0.5 g . FFPE samples should be transported at room temperature, while fresh tissue samples should be placed in tissue preservation solution and transported under low-temperature conditions. The availability of tissue samples will not affect patient enrollment.

[3] Each subject is required to provide 10 ml of whole blood and 10 ml of stool samples at the following time points for tumor biomarker analysis: before the first dose, at each imaging assessment during treatment (prior to the next treatment initiation), and at disease progression confirmation.

[4] Safety follow-up will be conducted 30 ± 7 days after the last dose or before the initiation of new anti-tumor therapy, whichever occurs first. All adverse events (AEs) occurring before the safety follow-up visit should be recorded until they resolve to Grade 0-1 or return to baseline levels, or if the investigator determines that further follow-up is unnecessary (e.g., if the event is irreversible or has sufficiently improved). Serious adverse events (SAEs) occurring within 90 days after the last dose or before the start of new anti-cancer treatment (whichever occurs first) will be followed up and documented.

[5] Survival follow-up: Conducted every 90 days (±7 days) after the safety visit, with telephone follow-up being acceptable.

[6] Radiotherapy should commence within one week after initiating targeted therapy ± sintilimab during the first cycle.
